# Estimates and Temporal Trend for US Nationwide 30-Day Hospital Readmission Among Patients With Ischemic and Hemorrhagic Stroke

**DOI:** 10.1001/jamanetworkopen.2018.1190

**Published:** 2018-08-17

**Authors:** Arvind B. Bambhroliya, John P. Donnelly, Eric J. Thomas, Jon E. Tyson, Charles C. Miller, Louise D. McCullough, Sean I. Savitz, Farhaan S. Vahidy

**Affiliations:** 1 Department of Neurology, The Institute for Stroke and Cerebrovascular Diseases, McGovern Medical School at The University of Texas Health Science Center at Houston (UTHealth); 2 Department of Epidemiology, University of Alabama School of Public Health, Birmingham; 3 Department of Internal Medicine, McGovern Medical School at The University of Texas Health Science Center at Houston (UTHealth); 4 Center for Clinical Research & Evidence-Based Medicine, McGovern Medical School at The University of Texas Health Science Center at Houston (UTHealth)

## Abstract

**Importance:**

Readmission reduction is linked to improved quality of care, saves cost, and is a desirable patient-centered outcome. Nationally representative readmission metrics for patients with stroke are unavailable to date. Such estimates are necessary for benchmarking performance.

**Objectives:**

To provide US nationwide estimates and a temporal trend for overall, planned, and potentially preventable 30-day hospital readmission among patients with ischemic and hemorrhagic stroke; to investigate the association between hospitals’ stroke discharge volume, teaching status, and 30-day readmission; and to highlight reasons for 30-day readmission and explore the association of 30-day readmission in terms of mortality, length of stay, and cost of care among patients with stroke.

**Design, Setting, and Participants:**

Cohort, year-wise analysis of the Nationwide Readmissions Database between January 1, 2010, and September 30, 2015. The setting was a population-based cohort study providing national estimates of 30-day readmission. The database represents 50% of all US hospitalizations from 22 geographically dispersed states. Participants were adult (≥18 years) patients with a primary discharge diagnosis of intracerebral hemorrhage, acute ischemic stroke, or subarachnoid hemorrhage. Hospitals were categorized by their annual stroke discharge volume and were classified as teaching hospitals if they had an American Medical Association–approved residency program or had a ratio of full-time equivalent interns and residents to beds of 0.25 or higher.

**Main Outcomes and Measures:**

Readmission was defined as any admission within 30 days of index hospitalization discharge. Using Centers for Medicare & Medicaid Services–defined algorithms, events were classified as planned or unplanned and as potentially preventable.

**Results:**

Based on study criteria, 2 078 854 eligible patients were included (mean [SE] age, 70.02 [0.07] years; 51.9% female). Thirty-day readmission was highest for patients with intracerebral hemorrhage (13.70%; 95% CI, 13.40%-13.99%), followed by patients with acute ischemic stroke (12.44%; 95% CI, 12.33%-12.55%) and patients with subarachnoid hemorrhage (11.48%; 95% CI, 11.01%-11.96%). On average, there was a 3.3% annual decline in readmission between 2010 and 2014, which was statistically significant for the period of investigation (odds ratio, 0.96; 95% CI, 0.95-0.97). Patients discharged from nonteaching hospitals with high stroke discharge volume were at a significantly higher risk of 30-day readmission, and the top 2 reasons for readmission were acute cerebrovascular disease and septicemia.

**Conclusions and Relevance:**

This study suggests that nationally representative readmission metrics can be used to benchmark hospitals’ performance, and a temporal trend of 3.3% may be used to evaluate the effectiveness of readmission reduction strategies.

## Key Points

**Question:**

What is the nationwide proportional change in 30-day hospital readmission among US patients with ischemic and hemorrhagic stroke between 2010 and 2015?

**Findings:**

In this 6-year population-based cohort study of approximately 2 million adult patients with stroke, 13.7% of patients with intracerebral hemorrhage, 12.4% of patients with acute ischemic stroke, and 11.5% of patients with subarachnoid hemorrhage were readmitted within 30 days. Among all patients with stroke, 30-day hospital readmission declined by an annual mean of 3.3% between 2010 and 2014.

**Meaning:**

It seems that national estimates can be used to benchmark performance, and a temporal trend of 3.3% may be used to evaluate the effectiveness of readmission reduction strategies among patients with stroke.

## Teaser

This population-based cohort study uses AHRQ National Readmission Database data to characterize trends in overall, planned, and potentially preventable 30-day hospital readmission rates among patients with ischemic and hemorrhagic stroke between 2010 and September 2015.

## Introduction

Readmission reduction is linked to improved quality of care and cost savings across health care systems in many countries, including the United States.^1^ Reduction of 30-day hospital readmission among patients with acute ischemic stroke (AIS) is a quality metric established by the Centers for Medicare & Medicaid Services (CMS) in the United States. However, despite the emphasis on tracking and reporting 30-day readmission for CMS beneficiaries with AIS, little is known about the nationwide proportion of patients with ischemic and hemorrhagic stroke readmitted within 30 days. Prior published reports of stroke-related readmission are exclusive to the CMS patient population 65 years or older and do not represent more recent data.^2,3,4^ Establishing contemporary, nationwide readmission metrics for various stroke subtypes among patients of all ages insured by all payer types (including the uninsured) is necessary to investigate the effectiveness of readmission reduction measures and is of interest to multiple stakeholders. In addition, quantifying the trend of stroke-related readmission is important to comprehensively evaluate the association of future readmission reduction strategies over time. It has also been reported that the overall burden of readmission is disproportionately borne by high-volume, academic hospitals.^5^ These health care facilities serve as safety-net hospitals in their communities and provide access to a wide case, payer, and population mix of patients. The population-level disproportionate burden across various facility types for stroke-related readmission has not been quantified to date. Finally, there is a dearth of information about the population-wide association of stroke-related readmission in terms of hospital outcomes and resource use relative to an early readmission. Understanding this association with resources and reasons for readmission will be helpful for constructing future preventive frameworks.

Our objective was to examine population-based, nationwide 30-day stroke-related readmission metrics over a recent 6-year period (between January 1, 2010, and September 30, 2015) stratified by stroke subtype. We hypothesized that there would be a significant reduction of 30-day stroke-related readmission during this period. We further aimed to investigate the association between hospitals’ stroke discharge volume (SDV) and their teaching status and readmission rate. We hypothesized that large-volume teaching hospitals would bear a disproportionate burden of stroke-related readmission. Finally, we explored whether readmitted patients have higher in-hospital mortality, longer length of stay (LOS), and higher costs compared with the mean of these metrics at index admission among patients with various stroke subtypes.

## Methods

### Design and Database

This was a population-based cohort study in which eligible patients with stroke were identified and followed up for 30-day hospital readmission during each individual year of the US Nationwide Readmissions Database between 2010 and 2015. The Nationwide Readmissions Database is part of the Health Care Utilization Project (HCUP) of the Agency for Healthcare Research and Quality (AHRQ).^6^ It comprises a nationally representative, weighted probability sample of approximately 36 million discharges per year from short-term hospitals of 22 geographically dispersed states in the United States (27 states were included for 2015). The nonweighted sample represents 50% of all US hospitalizations. Investigators received requisite training and conformed to the data use agreements with the HCUP. The HCUP databases conform to the definition of a limited data set; as per the data use agreement with the HCUP, review by an institutional review board is not required for use of limited data sets. The analysis and reporting followed the Strengthening the Reporting of Observational Studies in Epidemiology (STROBE) reporting guideline.^7^

### Target Patient Population and Case Definitions

We used *International Classification of Diseases*, *Ninth Revision* (*ICD-9*) codes to identify adults (≥18 years) with a primary diagnosis of ischemic stroke (codes 433.x1, 434.x1, and 436), primary intracerebral hemorrhage (ICH) (code 431), and subarachnoid hemorrhage (SAH) (code 430). Procedure codes were used to identify patients with stroke who received intravenous thrombolytics or endovascular treatment and underwent procedures, such as extraventricular drain placement, surgical decompression or hematoma evaluation, mechanical ventilation, and endotracheal or gastric tube placement. To avoid coding inconsistencies between *ICD-9* and *International Statistical Classification of Diseases and Related Health Problems, Tenth Revision*, data from the last quarter of 2015 were excluded. We also excluded same-day events, discharges in the last month of analysis (by each year), and in-hospital deaths (for index admission). For analysis of patients with hemorrhagic stroke (ICH or SAH), we excluded all patients who had a concurrent diagnosis of head trauma. Eligible patients had their first (index) hospital admission tagged for further analyses. Readmission was defined as any admission within 30 days of index hospitalization discharge. Using CMS-defined algorithms, events were classified as planned or unplanned, and potentially preventable readmissions attributable to ambulatory care–sensitive conditions were identified (eAppendix in the Supplement).^8^ For subanalyses, we also identified patients with the same primary discharge diagnosis on readmission as that of their index admission and evaluated reasons for readmission using the AHRQ’s Clinical Classification Software–based diagnostic categories.^9^

### Covariates, Subgroups, and Cost

We categorized hospitals based on their annual SDV as low (11-50), medium (51-175), high (176-350), or very high (>350). Hospitals with an SDV of 10 or less were excluded. Hospitals were classified as teaching hospitals if they had an American Medical Association–approved residency program or had a ratio of full-time equivalent interns and residents to beds of 0.25 or higher. For some analyses, we dichotomized the period of investigation as preimplementation and postimplementation of excess readmission penalties under the CMS’ Hospital Readmissions Reduction Program (HRRP) (discharges before vs on or after October 1, 2012).^10^ Costs were obtained by using the AHRQ’s ratios of cost to charge and were inflation adjusted for 2014 using the Chained Consumer Price Index for all urban consumers and medical care services from the US Bureau of Labor Statistics.^11^

### Statistical Analysis

We used survey design methods by taking into account sampling weights and clustering of discharges within hospitals to report national estimates of proportions and 95% CIs for overall, planned, and potentially preventable 30-day readmission during each individual year of analysis. Similar estimates are provided for readmission among patients discharged from teaching vs nonteaching hospitals and from hospitals with varying SDV. We fit survey design multivariable logistic regression models to assess the trend in annual proportion of 30-day readmission over the period of investigation and to investigate the association between CMS’ HRRP implementation and stroke-related readmission. Odds ratios (ORs) and 95% CIs are reported as estimates of likelihood of 30-day readmission across years and periods. Analyses controlled for potential changes in patients’ demographic, social, and comorbidity profiles. We also fit similar models to evaluate the association between stroke-related readmission and hospitals’ SDV and their teaching status and explored the interaction between SDV and teaching status for their association with readmission. Goodness-of-fit tests and area under the curve statistics were used to assess model fit and discrimination. With 252 406 events (30-day readmissions) in the combined data set, we had greater than 95% power to satisfy all of our analytical aims, including evaluation of interaction terms. Analyses were performed using statistical software (Stata, version 14; StataCorp LP).

## Results

### Analysis Population and Readmission Proportions

**Figure 1.**
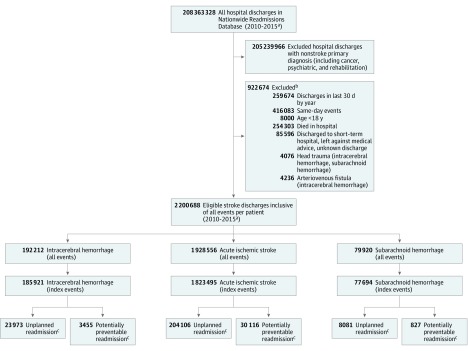
Eligible Stroke Discharges, Reasons for Exclusion, and Proportion of Overall, Unplanned, and Potentially Preventable Stroke-Related Nationwide 30-Day Readmissions Between January 1, 2010, and September 30, 2015 The proportion (95% CI) of 30-day overall readmissions among index events for acute ischemic stroke was 12.44% (12.33%-12.55%); intracerebral hemorrhage, 13.70% (13.40%-13.99%); and subarachnoid hemorrhage, 11.48% (11.01%-11.96%). The proportion (95% CI) of unplanned readmissions among 30-day readmissions for acute ischemic stroke was 90.00% (89.74%-90.26%); intracerebral hemorrhage, 94.15% (93.56%-94.70%); and subarachnoid hemorrhage, 90.63% (89.40%-91.73%). The proportion (95% CI) of potentially preventable readmissions among 30-day readmissions for acute ischemic stroke was 13.28% (13.03%-13.53%); intracerebral hemorrhage, 13.57% (12.82%-14.35%); and subarachnoid hemorrhage, 9.27% (8.26%-10.39%). ^a^Inclusive of data between January 1, 2010, and September 30, 2015. ^b^Reasons not mutually exclusive. ^c^Unplanned and potentially preventable readmissions not mutually exclusive.

The Nationwide Readmissions Database contains information on 208 363 328 hospital discharges between January 1, 2010, and September 30, 2015. Of these, 3 123 362 records were identified as having a primary discharge diagnosis of a stroke subtype (ICH, AIS, or SAH). Based on our criteria, 2 200 688 eligible stroke hospital discharges were included in our analyses. Of these, 87.6% were AIS, 8.7% were ICH, and 3.6% were SAH. Among the 2 078 854 index events (first event during each year) for all stroke subtypes, the mean (SE) patient age was 70.02 (0.07) years, and 51.9% were female. Summary data on demographics, hospital factors, comorbidity, disease severity, and treatment variables for the overall, readmitted, and nonreadmitted stroke discharges are listed in eTable 1 in the Supplement. Based on analysis of index events, 30-day readmission was highest for patients with ICH (13.70%; 95% CI, 13.40%-13.99%), followed by patients with AIS (12.44%; 95% CI, 12.33%-12.55%) and patients with SAH (11.48%; 95% CI, 11.01%-11.96%). More than 90% of all 30-day stroke-related readmissions were unplanned; depending on stroke subtype, up to 13.6% were deemed potentially preventable. Details on selection of eligible stroke discharges, index events, and stroke subtype–specific readmission metrics are shown in Figure 1.

### Trends in Nationwide Stroke-Related 30-Day Readmissions

**Figure 2.**
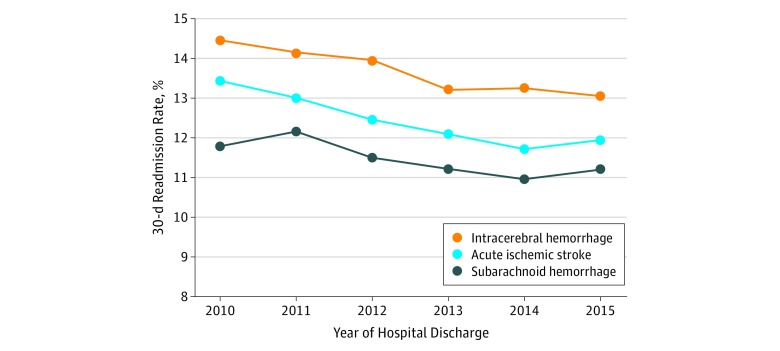
Proportion of Patients With Intracerebral Hemorrhage, Acute Ischemic Stroke, and Subarachnoid Hemorrhage Readmitted Within 30 Days of Index Hospital Discharge by Year of Investigation Adjusted odds ratios of 30-day readmissions by year were 0.96 (95% CI, 0.95-0.97) for intracerebral hemorrhage, 0.97 (95% CI, 0.95-0.98) for acute ischemic stroke, and 0.96 (95% CI, 0.94-0.99) for subarachnoid hemorrhage. Odd ratios were adjusted for the following: age, sex, insurance, patient location, median household income for patient’s zip code, Charlson Comorbidity Index, number of chronic conditions, atrial fibrillation, alcohol abuse, deficiency anemias, chronic blood loss anemia, congestive heart failure, coagulopathy, uncomplicated diabetes, diabetes with chronic complications, hypertension (combined uncomplicated and complicated), liver disease, fluid and electrolyte disorders, other neurological disorders, obesity, pulmonary circulation disorders, renal failure, solid tumor without metastasis, psychoses, depression, chronic pulmonary disease, drug abuse, peripheral vascular disorders, peptic ulcer disease (excluding bleeding), and valvular disease. Results shown for 2015 include data between January 1 and September 30, 2015.

The overall proportion of 30-day stroke-related readmission was highest in 2010 (13.47%; 95% CI, 13.19%-13.76%). On average, there was a 3.3% annual decline in 30-day readmission between 2010 and 2014. There was a statistically significant annual decline in likelihood of 30-day readmission by 4.0% over the period of investigation (OR, 0.96; 95% CI, 0.95-0.97) after controlling for potential changes in demographic, social, and comorbidity case mix across years. Our adjusted multivariable analyses further indicated that the probability of a nationwide 30-day stroke-related readmission was estimated to be 17.2% and 16.5% less likely during 2014 and 2015, respectively, compared with 2010 and 12.1% less likely after implementation of the CMS’ HRRP penalties compared with the preimplementation period. A similar decline in stroke-related readmission was observed across all stroke subtypes, with ICH readmissions remaining the highest across the analysis period. Figure 2 shows stroke subtype–specific proportion of 30-day readmission across the years of investigation, and the Table lists proportions, ORs, and 95% CIs for annual likelihood of 30-day readmission for all stroke subtypes, specific stroke subtypes, and before and after CMS’ HRRP implementation.

**Table.** Proportions, Odds Ratios, and 95% CIs for Year-Wise 30-Day Readmission Among Patients With All Stroke Subtypes, Intracerebral Hemorrhage, Acute Ischemic Stroke, and Subarachnoid Hemorrhage^a^

Variable	All Stroke Subtypes		Intracerebral Hemorrhage		Acute Ischemic Stroke		Subarachnoid Hemorrhage	
	Readmission, % (95% CI)	OR (95% CI)	Readmission, % (95% CI)	OR (95% CI)	Readmission, % (95% CI)	OR (95% CI)	Readmission, % (95% CI)	OR (95% CI)
**Year of Discharge**								
2010	13.47 (13.19-13.76)	1 [Reference]	13.44 (13.15-13.74)	1 [Reference]	14.46 (13.76-13.15)	1 [Reference]	11.79 (13.74-13.71)	1 [Reference]
2011	13.05 (12.72-13.39)	0.96 (0.92-0.99)	13.01 (12.68-13.36)	0.96 (0.88-1.05)	14.16 (13.39-12.68)	0.96 (0.92-1.00)	12.16 (13.36-13.41)	1.02 (0.86-1.22)
2012	12.52 (12.20-12.84)	0.91 (0.87-0.94)	12.45 (12.15-12.75)	0.95 (0.87-1.04)	13.97 (12.84-12.15)	0.91 (0.87-0.94)	11.49 (12.75-13.20)	0.96 (0.81-1.13)
2013	12.15 (11.93-12.37)	0.87 (0.84-0.90)	12.10 (11.87-12.32)	0.88 (0.81-0.96)	13.20 (12.37-11.87)	0.87 (0.84-0.90)	11.22 (12.32-12.55)	0.91 (0.77-1.06)
2014	11.79 (11.59-12.00)	0.83 (0.80-0.86)	11.71 (11.50-11.92)	0.87 (0.81-0.95)	13.25 (12.00-11.50)	0.83 (0.80-0.85)	10.96 (11.92-12.64)	0.87 (0.74-1.01)
2015	11.98 (11.79-12.18)	0.83 (0.81-0.86)	11.94 (11.73-12.14)	0.85 (0.78-0.93)	13.05 (12.18-11.73)	0.84 (0.81-0.86)	11.20 (12.14-12.38)	0.86 (0.73-1.00)
**Hospital Readmissions Reduction Program Status**								
Before the CMS’ HRRP^b^	13.08 (12.90-13.27)	1 [Reference]	14.25 (13.80-14.72)	1 [Reference]	13.04 (12.85-13.22)	1 [Reference]	11.91 (11.15-12.71)	1 [Reference]
After the CMS’ HRRP^c^	11.96 (11.85-12.08)	0.88 (0.86-0.90)	13.19 (12.83-13.57)	0.89 (0.85-0.94)	11.90 (11.78-12.02)	0.88 (0.86-0.90)	11.08 (10.57-11.60)	0.87 (0.80-0.96)

Abbreviations: CMS, Centers for Medicare & Medicaid Services; HRRP, Hospital Readmissions Reduction Program; OR, odds ratio.

^a^ Odds ratios were adjusted for the following: age, sex, insurance, patient location, median household income for patient’s zip code, Charlson Comorbidity Index, number of chronic conditions, atrial fibrillation, alcohol abuse, deficiency anemias, chronic blood loss anemia, congestive heart failure, coagulopathy, uncomplicated diabetes, diabetes with chronic complications, hypertension (combined uncomplicated and complicated), liver disease, fluid and electrolyte disorders, other neurological disorders, obesity, pulmonary circulation disorders, renal failure, solid tumor without metastasis, psychoses, depression, chronic pulmonary disease, drug abuse, peripheral vascular disorders, peptic ulcer disease (excluding bleeding), and valvular disease.

^b^ Defined as discharges before October 1, 2012, based on effective date of the CMS’ HRRP implementation.

^c^ Defined as discharges after October 1, 2012, based on effective date of the CMS’ HRRP implementation.

### Burden of Stroke-Related 30-Day Readmission Across Hospitals

**Figure 3.**
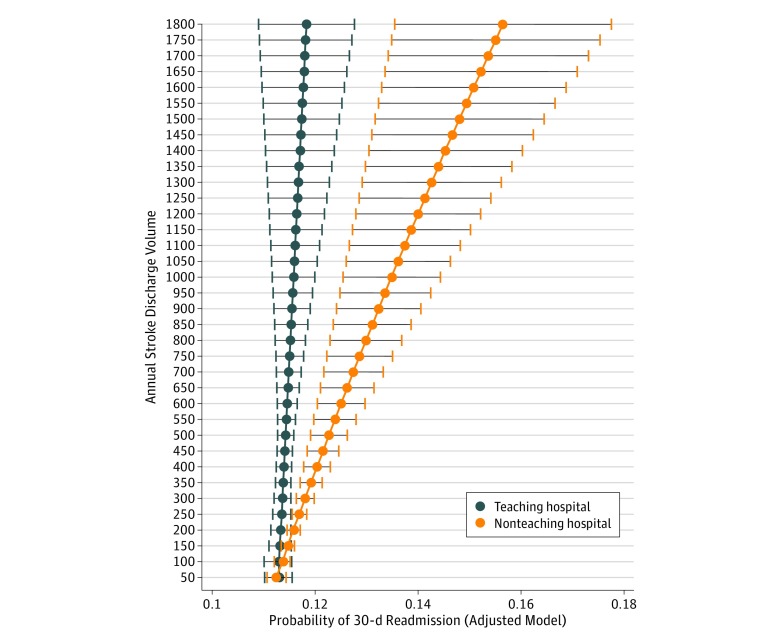
Probability and 95% CI of 30-Day Stroke-Related Readmission Based on Hospitals’ Stroke Discharge Volume in Increments of 50 for Teaching and Nonteaching Hospitals in the 2010 to 2015 Nationwide Readmissions Database Values on the x-axis were obtained from multivariable survey design models adjusted for patient case mix of demographic and comorbidity variables. The y-axis represents hospitals’ annual stroke discharge volume. Hospitals with stroke discharge volume of 10 or less were excluded. The maximum stroke discharge volume for nonteaching hospitals is 698. Data for nonteaching hospitals beyond the maximum are modeled for comparison with teaching hospitals.

A mean of 1397 hospitals per year were included in our analyses. The median annual SDV among these hospitals was 99 (interquartile range, 37-230). Our adjusted multivariable model indicated that higher SDV and nonteaching status were independently associated with higher stroke-related readmission for hospitals with very high vs low SDV (OR, 1.08; 95% CI, 1.03-1.12) and for nonteaching vs teaching hospitals (OR, 1.05; 95% CI, 1.03-1.07). We found a significant effect modification of teaching status and readmission by hospitals’ SDV. A higher SDV was significantly associated with 30-day stroke-related readmission among nonteaching hospitals (OR, 1.06; 95% CI, 1.02-1.11 for high vs low SVD and OR, 1.13; 95% CI, 1.07-1.18 for very high vs low SDV); however, a similar association was not observed for the 2 comparisons across teaching hospitals (2-sided *P* for interaction = .02 and .01, respectively) (eTable 2 and eFigure in the Supplement). We further characterized hospitals’ SDV based on increments of 50; after adjusting for demographic and comorbidity variables, there was a significantly increasing likelihood of 30-day stroke-related readmission for nonteaching hospitals with an annual SDV of 300 or higher compared with teaching hospitals with similar SDV (Figure 3).

### Reasons, Resources, and Outcomes for Stroke-Related Readmissions

**Figure 4.**
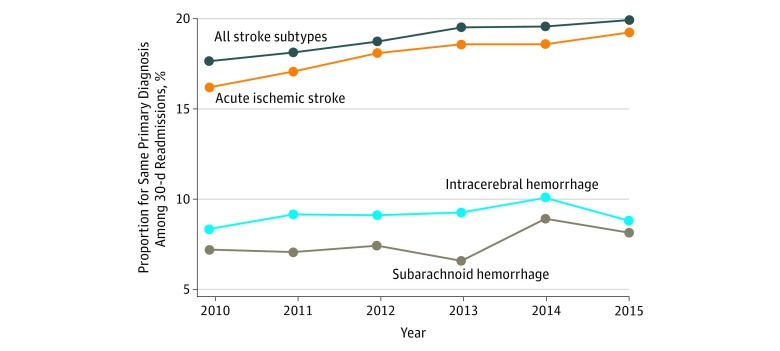
Year-Wise Proportion of 30-Day Hospital Readmission Among Patients With All Stroke Subtypes, Intracerebral Hemorrhage, Acute Ischemic Stroke, and Subarachnoid Hemorrhage With the Same Primary Diagnosis on Readmission as That on Index Hospitalization Results shown for 2015 include data between January 1 and September 30, 2015.

Among all 30-day stroke-related readmissions, 18.91% (95% CI, 18.64%-19.18%) had the same primary discharge diagnosis on readmission as that of their index hospitalization. This proportion was highest among patients with AIS (17.91%; 95% CI, 17.62%-18.19%), whereas it was 9.16% (95% CI, 8.52%-9.84%) for patients with ICH and 7.53% (95% CI, 6.68%-8.48%) for patients with SAH. Furthermore, this proportion increased over time, ranging from 17.66% (95% CI, 17.02%-18.32%) in 2010 to 19.94% (95% CI, 19.27%-20.62%) in 2015. The proportion of patients among the readmitted patients with the same primary diagnosis on readmission as that of their index admission for all stroke subtypes and for each stroke subtype is shown in Figure 4; eTable 3 in the Supplement lists proportions and 95% CIs for unplanned and potentially preventable readmissions by stroke subtype for each year among patients with the same readmission diagnosis as that of their index hospitalization. The analysis of readmission reasons yielded acute cerebrovascular disease and septicemia as the first and second leading reasons for readmission for all years and all stroke subtypes, accounting for 19.6% and 10.0%, respectively, of all readmissions. The top 25 reasons for readmission by year for each stroke subtype are listed in eTables 4, 5, 6, and 7 in the Supplement. On 30-day readmission, a higher proportion of patients with AIS experienced in-hospital mortality (6.54%; 95% CI, 6.36%-6.73%) compared with the proportion of patients who died during initial hospitalization (5.13%; 95% CI, 5.05%-5.22%). This was not observed for patients with ICH or SAH. The mean LOS on readmission was also longer for patients with AIS (6.50 days) compared with the mean index LOS (4.92 days). Likewise, the inflation-adjusted mean cost per stay was higher on readmission for patients with AIS ($10 881 vs $12 303 for index vs readmission). Comparative data between index hospitalization and readmission for mortality, LOS, and cost are listed in eTable 8 in the Supplement.

## Discussion

We present nationally representative readmission metrics during a contemporary 6-year period for patients with ICH, AIS, and SAH of all ages and all payer types. These analyses serve to address knowledge gaps in trends of readmission metrics and highlight that patients with stroke discharged from higher-volume nonteaching hospitals may be targeted for readmission reduction. These metrics are helpful for defining policy and practice and provide an estimate to measure the effectiveness of various readmission reduction strategies at the local level. To our knowledge, such data have not been previously reported for patients with stroke.

We report that a considerable proportion of patients with stroke are readmitted within 30 days of hospital discharge, with a significantly higher proportion among patients with ICH compared with patients with AIS (13.7% vs 12.4%). More than 90% of readmissions are unplanned, and up to 13% may be potentially preventable. Publicly reported readmission measures based on analyses of the CMS data are limited to patients with AIS who are 65 years or older. Our analyses of more than 2 million patients indicate that up to 35% of patients with stroke are younger than 65 years and that more than 25% were not insured by the CMS. Even among patients with AIS, the 30-day readmission was significantly lower for patients younger than 65 years compared with those 65 years or older (10.8% vs 13.3%). Furthermore, there were significant differences in readmission rates between payer types (OR, 0.70; 95% CI, 0.68-0.72 for private insurance vs Medicare) even after controlling for age. These data suggest that CMS estimates, although meaningful for CMS-insured elderly patients with AIS, may not be nationally representative and indicate that additional analyses and reporting of readmission metrics among patients of all stroke subtypes, all age groups, and all payer types are warranted to fill the knowledge gaps. Sole reliance on CMS measures to penalize hospitals for excess readmissions has also been previously questioned.^12^ Our analyses indicate that stroke-related readmission gradually declined between 2010 and 2014, with a mean annual reduction of 3.3% and a reduction of 12.5% between 2010 and 2014. A similar decline was not observed between 2014 and 2015, possibly because of the exclusion of data from the third quarter of 2015. However, CMS^13^ data for patients with AIS also suggest a steady readmission rate between 2013-2014 and 2014-2015. While there are no prior reports of nationwide trends among all stroke-related readmissions, our findings are comparable to gains in readmission reduction previously reported for other HRRP-targeted^14^ and HRRP-nontargeted conditions between 2007-2008 and 2014-2015. Furthermore, our data show a readmission reduction of 4.9% between 2012 and 2014 for patients with AIS. This estimate is comparable to a readmission reduction of 4.7% for patients with AIS in CMS data during a similar period.^13^ Also, the cumulative decline in readmission for 4 other high-volume conditions between 2009 and 2013 has been reported to be 5.4%.^15^ Therefore, we believe that an annual 3.3% decline in stroke-related readmission can be used to guide and monitor the effectiveness of stroke-related readmission reduction programs.

In addition to the absence of stroke-related readmission reduction programs at the national level, there could be multiple other reasons for the nationwide decline. First, patients with stroke are elderly with multiple comorbidities, particularly cardiovascular comorbidities. Because our analyses include all-cause readmissions, it is likely that readmission reduction among patients with stroke is corollary to the overall readmission reduction reported for patients without stroke. It is important to highlight that 30-day readmission attributed to the same primary diagnosis as that of the index admission did not show a decreasing trend. Second, it is also possible that an emphasis on improving postacute transition of care (TOC) has accounted for some of this decline.^16^ Multiple regional and local initiatives are under way to improve hospital discharge processes.^17,18,19,20,21^ However, there remains considerable lack of standardization in TOC models across the nation for patients with stroke, and the overall influence of TOC improvement on stroke-related readmission remains to be systematically demonstrated.^22^ Third, it is also likely that stroke-related readmission metrics have been influenced by well-documented effects of measuring and reporting health care quality.^23^

Our adjusted analyses indicate that likelihood of readmission significantly increases with higher SDV for nonteaching hospitals, but a similar outcome is not observed for teaching institutions. Although seemingly small, these associations translate into a population-based increased risk of readmission among patients with stroke at nonteaching hospitals. There is a growing concern that various quality programs, including the HRRP, will disproportionately penalize safety-net hospitals.^24^ It has also been reported that large hospitals and teaching hospitals had a significantly higher likelihood of penalization under the HRRP.^25^ However, a prior analysis of CMS data from 2006 found no differences in readmission rates between patients with stroke treated at primary stroke centers and those discharged from noncertified centers.^26^ This is important because many high-volume teaching hospitals serve as safety-net hospitals for their communities, catering to the health care needs of the uninsured and underinsured. The effect modification by hospitals’ teaching status is likely explained by better adherence to quality-of-care metrics at these centers, including an emphasis on TOC variables. For example, many teaching hospitals have adjoining outpatient clinics or are staffed by faculty who also attend in an ambulatory clinic. Certain reports indicate better adherence to in-hospital quality of care and improved outcomes for patients discharged from hospitals contributing data to national stroke registries.^27,28^ Such improvements specifically include greater proportions of patients with AIS receiving thrombolytic therapies and shorter door-to-needle times. Significantly lower rates of 30-day readmission were previously demonstrated among patients with AIS who received thrombolytic therapies.^29^ Improved acute treatment metrics have also been strongly attributed to the use of telestroke technology and organization of care delivery.^30,31^ It is likely that a larger number of teaching hospitals are reporting their data to national registries and are regional telestroke centers and thus consistently provide better quality of care to patients with stroke even at higher volumes. Identifying specific patient populations with stroke at higher risk of readmission are much needed for practice and policy decisions.

We examined reasons for readmission using 2 different approaches and consistently found that approximately 1 in 5 readmissions were attributable to acute cerebrovascular disease or had the same principal diagnosis as that of the index hospitalization. The second most prevalent reason for readmission was septicemia. These findings were consistent across all years of analysis for all stroke subtypes. Our findings verify prior individual reports and systematic reviews regarding reasons for readmission among patients with stroke.^2,3,29,32^ These findings highlight the importance of secondary stroke prevention and warrant a deeper examination of various mechanistic factors that mediate early readmission in patients with stroke. We also report that patients with AIS who experience 30-day readmission have greater mortality, longer LOS, and higher cost per stay on readmission compared with the mean of these metrics on index admission. It has been previously shown that readmission is costlier compared with the index admission for a several other high-volume conditions as well.^15^ We believe that excess cost on readmission is mostly explained by longer LOS and is likely attributable to the need for higher intensity of care. Further analyses are warranted to elucidate factors that lead to excess resource use and mortality among patients with AIS on 30-day readmission.

### Limitations

Our analyses should be interpreted in light of the following limitations. First, the use of an administrative database did not allow us to control for stroke-specific severity measures. However, a large sample size rendered enough power to adjust for an extensive set of comorbidity and other sociodemographic variables and adequately test for effect modification. Second, the design of the Nationwide Readmissions Database did not enable us to track individual patients across multiple years. Therefore, our analyses represent cross-sectional estimates for each included year. Likewise, the readmission reduction reported across the period of CMS’ HRRP implementation does not convey a direct influence of the HRRP on stroke-related readmission. Third, potentially preventable readmissions were defined based on CMS methods for analyses of administrative data. Reasons for readmission are multifactorial (patient, quality of care, social, and access to care); therefore, classification of a readmission as potentially preventable requires deeper, prospective, contextual analyses. Until such time as these factors are clearly elucidated, using readmission reduction as a primary outcome in clinical trials will remain challenging. Fourth, the use of *ICD-9* codes to identify cases may have been sensitive to variations in coding practices across various settings or over time. Such variations may introduce bias in risk-adjustment models or case ascertainment. However, sensitivity, specificity, and positive predictive value of *ICD-9* codes for stroke have been reported to be high.^33,34,35,36,37^ Furthermore, we did not include data from the last quarter of 2015 to avoid the possible influence of changes in coding practices. While our work may address certain limitations of existing CMS readmission measures, it does not completely eliminate concerns raised in the literature regarding the broad use of hospital readmission as a quality measure for hospital rankings.^12^ Although nationally derived, our estimates are best suited for understanding and improving care at a local level, rather than establishing a national performance standard. Further work is warranted to establish the validity of national readmission measures, particularly in the context of emerging evidence of compromised survival associated with readmission reduction for other conditions.^38^

## Conclusions

We provide novel and contemporary evidence of declining 30-day readmission among patients with all stroke subtypes. This decline seems to be explained largely by the overall decline in 30-day readmission for other high-volume conditions. We further identify nonteaching hospitals with higher SDV as potential targets for improvement in stroke-related readmission, perhaps focused on secondary stroke prevention. We provide an estimate of a temporal trend as a metric for planning and evaluation of readmission reduction strategies at the level of individual hospitals. However, patient-level and health care environment–specific prospective research is needed to fully understand reasons for readmission and development of targeted readmission reduction strategies among patients with stroke.

## Notes

Supplement. **eAppendix.** Supplemental Methods **eTable 1.** Descriptive Univariable Analysis of the Overall, Readmitted, and Non-Readmitted Index Stroke Discharges in the National Readmission Database (January 2010-September 2015) **eTable 2.** Multivariate Analyses for Association Between 30-Day Readmission and Hospital Teaching Status and Stroke Discharge Volume **eTable 3.** Proportion and 95% CI for Same Primary Diagnosis, Unplanned, and Preventable Readmissions Among Readmitted Patients by Year for Stroke Subtypes **eTable 4.** Top 25 Causes of 30-Day Readmission for All Stroke Subtypes by Year **eTable 5.** Top 25 Causes of 30-Day Readmission for Ischemic Stroke by Year **eTable 6.** Top 25 Causes of 30-Day Readmission for Intracerebral Hemorrhage by Year **eTable 7.** Top 25 Causes of 30-Day Readmission for Subarachnoid Hemorrhage by Year **eTable 8.** Comparison of Length of Stay, In-hospital Mortality, and Cost of Care for Ischemic and Hemorrhagic Stroke by Year **eFigure.** Probability and 95% CI (Y-Axis) for 30-Day Stroke Related Readmissions for Hospitals With Varying Stroke Discharge Volume (X-Axis) Based on Hospitals’ Teaching Status **eReferences**
