# Supplementary material for: Estimates and Temporal Trend for US Nationwide 30-Day Hospital Readmission Among Patients With Ischemic and Hemorrhagic Stroke
Source: JAMA Netw Open. 2018 Aug 17;1(4):e181190. doi: 10.1001/jamanetworkopen.2018.1190 (PMC6324273; doi:10.1001/jamanetworkopen.2018.1190)
Supplement: Supplement. — eAppendix. Supplemental Methods eTable 1. Descriptive Univariable Analysis of the Overall, Readmitted, and Non-Readmitted Index Stroke Discharges in the National Readmission Database (January 2010-September 2015) eTable 2. Multivariate Analyses for Association Between 30-Day Readmission and Hospital Teaching Status and Stroke Discharge Volume eTable 3. Proportion and 95% CI for Same Primary Diagnosis, Unplanned, and Preventable Readmissions Among Readmitted Patients by Year for Stroke Subtypes eTable 4. Top 25 Causes of 30-Day Readmission for All Stroke Subtypes by Year eTable 5. Top 25 Causes of 30-Day Readmission for Ischemic Stroke by Year eTable 6. Top 25 Causes of 30-Day Readmission for Intracerebral Hemorrhage by Year eTable 7. Top 25 Causes of 30-Day Readmission for Subarachnoid Hemorrhage by Year eTable 8. Comparison of Length of Stay, In-hospital Mortality, and Cost of Care for Ischemic and Hemorrhagic Stroke by Year eFigure. Probability and 95% CI (Y-Axis) for 30-Day Stroke Related Readmissions for Hospitals With Varying Stroke Discharge Volume (X-Axis) Based on Hospitals’ Teaching Status eReferences [file jamanetwopen-1-e181190-s001.pdf]

Supplementary Online Content

Bambhroliya AB, Donnelly JP, Thomas EJ, et al. Estimates and temporal trend for US nationwide 30-day hospital readmission among patients with ischemic and hemorrhagic stroke. *JAMA Netw Open*. 2018;1(4):e181190. doi:10.1001/jamanetworkopen.2018.1190

eAppendix. Supplemental Methods

eTable 1. Descriptive Univariable Analysis of the Overall, Readmitted, and Non-Readmitted Index Stroke Discharges in the National Readmission Database (January 2010-September 2015)

eTable 2. Multivariate Analyses for Association Between 30-Day Readmission and Hospital Teaching Status and Stroke Discharge Volume

eTable 3. Proportion and 95% CI for Same Primary Diagnosis, Unplanned, and Preventable Readmissions Among Readmitted Patients by Year for Stroke Subtypes

eTable 4. Top 25 Causes of 30-Day Readmission for All Stroke Subtypes by Year

eTable 5. Top 25 Causes of 30-Day Readmission for Ischemic Stroke by Year

eTable 6. Top 25 Causes of 30-Day Readmission for Intracerebral Hemorrhage by Year

eTable 7. Top 25 Causes of 30-Day Readmission for Subarachnoid Hemorrhage by Year

eTable 8. Comparison of Length of Stay, In-hospital Mortality, and Cost of Care for Ischemic and Hemorrhagic Stroke by Year

eFigure. Probability and 95% CI (Y-Axis) for 30-Day Stroke Related Readmissions for Hospitals With Varying Stroke Discharge Volume (X-Axis) Based on Hospitals' Teaching Status

eReferences

This supplementary material has been provided by the authors to give readers additional information about their work.

©2018 Bambhroliya AB et al. *JAMA Network Open*

eAppendix. Supplemental Methods

Criteria to Define Stroke Sub-Types

We used International Classification of Disease – Ninth Revision (ICD-9) [1] codes to identify stroke sub-types and procedures. We used ICD-9 codes (433.01, 433.11, 433.21, 433.31, 433.81, 433.91, 434.01, 434.11, 434.91, and 436) to identify ischemic stroke cases. These codes have a sensitivity of 75%,[2] specificity of 95%,[2] and positive predictive value of 85-94%.[2-5] We used ICD-9 code (431) to identify intracerebral hemorrhage cases. This code has a sensitivity of 85%,[2] specificity of 96%,[2] and positive predictive value of 89-97%.[2,4,6] We used ICD-9 code (430) to identify sub arachnoid hemorrhage cases. This code has a sensitivity of 90%,[2] specificity of 97%,[2] and positive predictive value of 94-100%.[2,4,6]

Criteria to Identify Planned versus Unplanned Readmissions

We categorized readmissions as planned based on the Center for Medicare & Medicaid Services (CMS) definition of planned readmissions.[7] Planned versus unplanned readmissions are defined as: (1) an admission always considered as planned (obstetrical delivery, transplant surgery, maintenance chemotherapy, rehabilitation) is planned; (2) a non-acute readmission for a schedules procedure is planned; and (3) an admission for acute illness or for complications of care is unplanned. Diagnosis and procedure codes that are always considered planned, and procedure codes that are considered planned when they are not accompanied by acute diagnosis are available at the CMS Measure Information Form for the 30-day All-Cause Hospital Readmission measure (https://www.cms.gov/Medicare/Medicare-Fee-for-Service-Payment/PhysicianFeedbackProgram/Downloads/2014-ACR-MIF.pdf).

Criteria to Identify Potentially Preventable Readmissions

We categorized readmissions as potentially preventable based on the Agency for Healthcare Research and Quality (AHRQ) definition of Prevention Quality Indicators (PQIs).[8] PQIs are a set of measures that use hospital inpatient discharge data to identify ambulatory care-sensitive conditions (ACSCs) - conditions for which optimal outpatient care can potentially prevent the need for hospitalization, or for which early intervention can prevent complications or more severe disease. Our analyses included PQIs for adult admissions for diabetes (with short-term complications - PQI 01, long-term complications – PQI 03, uncontrolled diabetes PQI 14, or lower-extremity amputations PQI 16), chronic obstructive pulmonary disease – PQI 05, asthma – PQI 05 and PQI 15, hypertension – PQI 07, congestive heart failure – PQI 08, dehydration – PQI 10, bacterial pneumonia – PQI 11, urinary tract infection – PQI 12, and angina without procedure – PQI 13. We used validated codes from the International Classification of Diseases, Ninth Revision (ICD-9)[1] to identify visits related to these 12 PQIs. This analysis used version 5.0 of AHRQ's PQIs Technical Specifications) which include details of these PQIs, including ICD-9 codes used to identify each condition (http://www.qualityindicators.ahrq.gov/Archive/PQI_TechSpec_ICD9_v50.aspx).

Criteria to Identify Procedures

We used ICD-9 code (99.10) to identify cases who received intravenous thrombolytics. This code has a sensitivity of 55%, specificity of 98%, and positive predictive value of 84%.[9] Moradiya et al. reported that ICD-9 code 99.10 identified 73.5% of pharmacy billing–verified total thrombolytic cases.[10] We used ICD-9 codes for craniotomy (01.24), and craniectomy (01.25) codes to identify cases underwent surgical decompression. Dasenbrock et al. reported that procedure codes for craniotomy (01.24), craniectomy (01.25), and lobectomy (01.39, 01.53, 01.59) for cerebral infarction (433.11, 434.01, 434.11, and 434.91) had a sensitivity of 97.8%, specificity of 99.9%, positive predictive value of 88.2%, and negative predictive value of 99.9%.[11] We used ICD-9 codes to identify cases who received invasive mechanical ventilation (96.70), noninvasive mechanical ventilation (93.90), endotracheal tube placement (31.1, 31.2, 31.21, and 31.29) and gastric tube placement (43.11). Positive predictive values for mechanical ventilation and endotracheal placement had been reported as 93% and 100%, respectively.[12] Chiò et al reported that sensitivity was 92.3% for gastric tube placement (43.11), 78.3% for noninvasive mechanical ventilation (93.90), and 100% for tracheostomy (31.1) and positive predictive value of 100% for all three procedures in a population based study for amyotrophic lateral sclerosis.[13] It is unlikely that these procedure codes would be inaccurate as they are associated with high levels of reimbursement.[14] We used ICD-9 code (02.21) to identify extra-

ventricular drain placement as reported by Moradiya et al.[15] No prior study has validated this code for ascertainment of cases who received ventricular drain placement in stroke patients.

eTable 1. Descriptive Univariable Analysis of the Overall, Readmitted, and Non-Readmitted Index Stroke Discharges in the National Readmission Database (January 2010-September 2015)

	First Stroke Events in Analysis Population (N = 2,078,854)	Non-Readmitted (N = 1,819,132)	Readmitted (N = 259,722)	OR (95% CI)
**Demographic Characteristics**				
Age – mean (SE)	70.02 (0.07)	69.84 (0.07)	71.23 (0.09)	1.01 (1.01 – 1.01)
Female (%)	51.90	51.98	51.40	0.98 (0.96-0.99)
Insurance (%)				
Medicare	65.62	64.81	71.29	Ref
Medicaid	7.92	7.83	8.56	0.99 (0.97-1.02)
Private	18.49	19.06	14.55	0.69 (0.68-0.71)
Other	7.97	8.31	5.60	0.61 (0.59-0.63)
Patient County (%)				
“Central” Large Metro	24.65	24.32	27.03	Ref
“Fringe” Large Metro	25.57	25.52	25.95	0.91 (0.89-0.94)
Other (Non-Large Metro)	49.77	50.16	47.02	0.84 (0.83-0.86)
Median Household Income for Patient ZIP Code (Quartile)				
$1-37,999	31.49	31.29	32.89	Ref
$38,000-47,999	25.68	25.76	25.17	0.93 (0.91-0.95)
$48,000-63,999	23.30	23.37	22.8	0.93 (0.91-0.95)
≥$64,000	19.53	19.58	19.14	0.93 (0.91-0.95)
**Hospital Factors**				
Stroke Volume based Hospital Category				
11-50 Stroke Discharges	6.23	6.28	5.83	Ref
51-175 Stroke Discharges	22.51	22.53	22.35	1.07 (1.03-1.11)
176-350 Stroke Discharges	29.52	29.47	29.87	1.09 (1.06-1.13)
>=351 Stroke Discharges	41.75	41.72	41.95	1.08 (1.04-1.13)
Teaching Status				
Metropolitan Teaching	54.99	55	54.88	Ref
Metropolitan Non-Teaching / Non-Metropolitan	45.01	45	45.12	1.01 (0.99-1.02)
Hospital Bed size				
Small	11.02	11.08	10.62	Ref
Medium	24.44	24.51	23.95	1.02 (0.99-1.05)
Large	64.53	64.41	65.43	1.06 (1.03-1.09)
Hospital urban-rural designation				
Large metropolitan areas with at least 1 million residents	53.18	52.83	55.62	Ref
Small metropolitan areas with less than 1 million residents	36.51	36.75	34.86	0.90 (0.88-0.92)
Micropolitan areas	7.56	7.65	6.96	0.86 (0.83-0.89)
Not metropolitan or micropolitan (non-urban residual)	2.75	2.78	2.56	0.87 (0.83-0.92)
**Comorbidity and Disease Severity**				
Number of chronic conditions – mean (SE)	6.95 (0.02)	6.85 (0.02)	7.67 (0.02)	1.10 (1.10-1.10)
Charlson Comorbidity Index				

©2018 Bambhroliya AB et al. *JAMA Network Open*

	First Stroke Events in Analysis Population (N = 2,078,854)	Non-Readmitted (N = 1,819,132)	Readmitted (N = 259,722)	OR (95% CI)
1	25.91	27.92	11.83	Ref
≥ 2	74.09	72.08	88.17	2.89 (2.82-2.95)
Atrial Fibrillation	22.19	21.65	25.95	1.27 (1.25-1.29)
Hypertension	81.43	81.26	82.60	1.09 (1.07-1.12)
Coagulopathy	3.37	3.20	4.57	1.45 (1.40-1.51)
Congestive Heart Failure	12.76	12.04	17.78	1.58 (1.55-1.61)
Valvular disorders	9.13	8.94	10.49	1.19 (1.16-1.22)
Peripheral Vascular Disease	8.85	8.54	10.95	1.32 (1.29-1.35)
Disorder of pulmonary circulation	3.25	3.07	4.54	1.50 (1.45-1.56)
Chronic Pulmonary Disease	14.53	14.04	17.95	1.34 (1.31-1.37)
Chronic Blood loss	0.36	0.33	0.54	1.64 (1.48-1.82)
Anemia	12.15	11.41	17.30	1.62 (1.59-1.66)
Ulcer	0.02	0.02	0.04	1.84 (1.27-2.67)
Diabetes Mellitus	28.11	27.76	30.56	1.15 (1.13-1.16)
Diabetes with complications	6.09	5.75	8.47	1.52 (1.48-1.56)
Liver Disease	1.30	1.22	1.84	1.52 (1.44-1.60)
Renal Failure	13.60	12.73	19.69	1.68 (1.65-1.71)
Fluid and electrolyte disorders	22.01	21.22	27.52	1.41 (1.39-1.43)
Psychoses	3.47	3.33	4.40	1.34 (1.29-1.39)
Depression	10.00	9.89	10.79	1.10 (1.08-1.13)
Other neurologic disorder	0.96	0.88	1.56	1.79 (1.68-1.91)
Drug Abuse	2.73	2.75	2.66	0.97 (0.93-1.01)
Alcohol	4.54	4.58	4.21	0.91 (0.88-0.95)
Obesity	9.87	9.88	9.77	0.99 (0.96-1.01)
All Patient Refined DRG severity of illness (Loss of function)				
Minor	12.76	13.47	7.80	Ref
Moderate	47.95	49.01	40.58	1.43 (1.39-1.47)
Major	31.34	30.30	38.61	2.20 (2.13-2.27)
Extreme	7.95	7.22	13.01	3.11 (2.99-3.24)
All Patient Refined DRG mortality (Likelihood of dying)				
Minor	29.24	30.63	19.52	Ref
Moderate	44.88	45.00	44.08	1.54 (1.50-1.57)
Major	18.81	17.78	25.94	2.29 (2.23-2.35)
Extreme	7.07	6.59	10.45	2.49 (2.40-2.59)
**Treatment Variables (%)**				
Intravenous tPA	5.64	5.67	5.45	0.96 (0.93-0.99)
Intra-arterial Therapy	0.80	0.79	0.90	1.15 (1.06-1.25)
Extra-ventricular Drain Placement	1.05	1.02	1.26	1.24 (1.16-1.33)
Hemicraniectomy/Hemicraniotomy	0.31	0.28	0.50	1.80 (1.60-2.02)
Invasive Ventilation	0.01	0.01	0.02	3.04 (1.50-6.15)
Noninvasive Ventilation	0.75	0.71	1.07	1.51 (1.41-1.62)
Tracheostomy	1.27	1.16	2.05	1.79 (1.69-1.89)
PEG Tube Placement	4.80	4.10	9.70	2.51 (2.44-2.59)

OR, odds ratio; CI, confidence interval; SE, standard error; Ref, reference category; PEG, percutaneous endoscopic gastrostomy.

eTable 2. Multivariate Analyses for Association Between 30-Day Readmission and Hospital Teaching Status and Stroke Discharge Volume

	Odds Ratios (95% CI) for 30-Day Stroke Readmission				
	Sub model 1 Overall without Teaching Status	Sub model 2 Overall with Teaching Status	Sub model 3 Only for Teaching	Sub model 4 Only for Non-Teaching	P value[a] for interaction
**Model 0: Hospital Category + Teaching Status**					
**Hospital Stroke Discharge Volume Category**					
11-50 Stroke Discharges	Ref				
51-175 Stroke Discharges	1.07 (1.03-1.11)	1.08 (1.04-1.12)	1.01 (0.9-1.14)	1.07 (1.03-1.11)	0.379
176-350 Stroke Discharges	1.09 (1.06-1.13)	1.11 (1.07-1.15)	1.00 (0.89-1.13)	1.12 (1.07-1.16)	0.091
>=351 Stroke Discharges	1.08 (1.04-1.13)	1.11 (1.07-1.16)	1.00 (0.89-1.13)	1.16 (1.11-1.22)	0.021
**Hospital Teaching Status**					
Teaching		Ref			
Non-Teaching		1.03 (1.01-1.05)			
**Model 1: Hospital Category + Teaching Status + Demographics[b]**					
**Hospital Stroke Discharge Volume Category**					
11-50 Stroke Discharges	Ref				
51-175 Stroke Discharges	1.06 (1.02-1.10)	1.07 (1.03-1.11)	0.99 (0.88-1.12)	1.07 (1.03-1.12)	0.225
176-350 Stroke Discharges	1.09 (1.04-1.12)	1.09 (1.05-1.13)	0.98 (0.87-1.11)	1.11 (1.06-1.15)	0.065
>=351 Stroke Discharges	1.09 (1.05-1.13)	1.12 (1.07-1.16)	1.01 (0.89-1.13)	1.16 (1.10-1.22)	0.032
**Hospital Teaching Status**					
Teaching		Ref			
Non-Teaching		1.04 (1.02-1.06)			
**Model 2 : Hospital Category + Teaching Status + Demographics + Comorbidities[c]**					
**Hospital Stroke Discharge Volume Category**					
11-50 Stroke Discharges	Ref				
51-175 Stroke Discharges	1.03 (0.99-1.07)	1.04 (1.00-1.08)	0.96 (0.84-1.09)	1.04 (1.00-1.09)	0.204
176-350 Stroke Discharges	1.03 (0.99-1.06)	1.04 (1.00-1.08)	0.91 (0.81-1.04)	1.06 (1.02-1.11)	0.024
>=351 Stroke Discharges	1.04 (1.00-1.08)	1.08 (1.03-1.12)	0.94 (0.83-1.07)	1.13 (1.07-1.18)	0.011
**Hospital Teaching Status**					
Teaching		Ref			
Non-Teaching		1.05 (1.03-1.07)			

CI, confidence interval; Ref, reference category. Sub models 3 and 4 are stratified models by teaching status of hospital.

[a] p value comparing ORs between Teaching and Non-Teaching using test command which uses adjusted Wald test

[b] Variables include age, gender, insurance, patient location, median household income for patient's ZIP Code

[c] Variables include age, gender, insurance, patient location, median household income for patient's ZIP Code, Charlson co-morbidity score, number of chronic conditions, atrial fibrillation, Alcohol abuse, Deficiency anemias, Chronic blood loss anemia, Congestive heart failure, Coagulopathy, , Diabetes, uncomplicated, Diabetes with chronic complications, Hypertension (combine uncomplicated

©2018 Bambhroliya AB et al. *JAMA Network Open*

and complicated), Liver disease, Fluid and electrolyte disorders, Other neurological disorders, Obesity, Pulmonary circulation disorders, Renal failure, Solid tumor without metastasis. Following variables were removed from model 2:
psychoses, depression, chronic pulmonary disease, drug abuse, peripheral vascular disorders, peptic ulcer disease excluding bleeding, valvular disease.

©2018 Bambhroliya AB et al. *JAMA Network Open*

eTable 3. Proportion and 95% CI for Same Primary Diagnosis, Unplanned, and Preventable Readmissions Among Readmitted Patients by Year for Stroke Subtypes

	Same Primary Diagnosis as Index Admission	Unplanned Readmissions	Preventable Readmissions
**All stroke sub-types**			
2010	17.66 (17.02-18.32)	90.82 (90.22-91.40)	14.06 (13.45-14.70)
2011	18.13 (17.44-18.83)	90.75 (90.20-91.28)	13.84 (13.27-14.42)
2012	18.74 (18.09-19.41)	90.31 (89.64-90.95)	13.34 (12.77-13.93)
2013	19.52 (18.90-20.15)	90.03 (89.43-90.59)	12.90 (12.35-13.47)
2014	19.58 (18.93-20.24)	89.94 (89.40-90.45)	12.59 (12.08-13.12)
2015	19.94 (19.27-20.62)	90.57 (90.02-91.09)	12.02 (11.47-12.59)
**Ischemic Stroke**			
2010	16.20 (15.58-16.84)	90.37 (89.68-91.02)	14.19 (13.55-14.86)
2011	17.08 (16.35-17.83)	90.39 (89.78-90.97)	14.01 (13.39-14.65)
2012	18.11 (17.42-18.82)	89.81 (89.08-90.50)	13.30 (12.70-13.93)
2013	18.59 (17.93-19.28)	89.55 (88.91-90.16)	12.89 (12.30-13.50)
2014	18.59 (17.93-19.26)	89.67 (89.10-90.22)	12.87 (12.33-13.43)
2015	19.25 (18.53-19.99)	90.24 (89.66-90.80)	12.13 (11.56-12.72)
**Intracerebral Hemorrhage**			
2010	8.36 (7.07-9.85)	94.25 (92.38-95.69)	14.12 (12.39-16.04)
2011	9.16 (7.61-10.98)	93.77 (92.16-95.07)	13.93 (12.26-15.78)
2012	9.12 (7.56-10.96)	94.69 (93.32-95.80)	14.44 (12.38-16.78)
2013	9.28 (7.95-10.80)	94.54 (93.32-95.55)	14.39 (12.77-16.18)
2014	10.08 (8.57-11.83)	93.53 (92.24-94.61)	11.77 (10.33-13.38)
2015	8.81 (7.36-10.52)	94.11 (92.09-95.64)	12.48 (10.39-14.92)
**Sub-Arachnoid Hemorrhage**			
2010	7.22 (5.21-9.92)	92.54 (90.08-94.43)	9.94 (7.92-12.41)
2011	7.08 (5.46-9.14)	92.02 (89.48-93.98)	9.39 (7.49-11.71)
2012	7.43 (5.66-9.69)	91.39 (87.37-94.22)	10.87 (7.69-15.14)
2013	6.59 (4.95-8.74)	89.55 (86.02-92.27)	9.38 (7.40-11.83)
2014	8.92 (6.62-11.92)	87.91 (84.55-90.61)	7.07 (5.46-9.11)
2015	8.15 (5.86-11.23)	90.10 (86.97-92.54)	8.79 (6.31-12.10)

©2018 Bambhroliya AB et al. *JAMA Network Open*

eTable 4. Top 25 Causes of 30-Day Readmission for All Stroke Subtypes by Year

Description	2010-2015Q3		2010		2011		2012		2013		2014		2015	
	Rank	%	Rank	%	Rank	%	Rank	%	Rank	%	Rank	%	Rank	%
Acute cerebrovascular disease	1	19.75	1	18.44	1	19.00	1	19.38	1	20.47	1	20.62	1	20.65
Septicemia (except in labor)	2	8.59	2	7.09	2	7.71	2	7.92	2	8.54	2	9.69	2	10.40
Occlusion or stenosis of pre-cerebral arteries	3	4.51	3	4.25	3	4.31	3	4.42	3	4.78	3	4.76	3	4.47
Acute and unspecified renal failure	4	3.13	7	2.75	5	3.11	5	2.97	4	3.42	4	3.29	4	3.32
Urinary tract infections	5	3.03	4	3.49	4	3.37	4	3.19	6	2.72	5	2.82	7	2.57
Cardiac dysrhythmias	6	2.78	6	3.04	8	2.67	6	2.92	5	2.88	7	2.52	6	2.61
Aspiration pneumonitis; food/vomitus	7	2.70	5	3.33	6	2.91	8	2.73	8	2.47	9	2.33	8	2.38
Congestive heart failure; non-hypertensive	8	2.69	8	2.65	7	2.71	9	2.66	7	2.61	6	2.77	5	2.66
Pneumonia (except that caused by tuberculosis or sexually transmitted disease)	9	2.41	9	2.51	9	2.53	7	2.80	11	2.29	11	2.12	11	2.12
Late effects of cerebrovascular disease	10	2.39	11	2.26	10	2.48	11	2.52	10	2.33	8	2.51	9	2.32
Transient cerebral ischemia	11	2.30	10	2.44	11	2.38	10	2.55	12	2.24	12	2.08	12	2.08
Gastrointestinal hemorrhage	12	2.20	12	2.03	12	2.13	12	2.01	9	2.35	10	2.31	10	2.29
Other nervous system disorders	13	1.72	15	1.61	15	1.65	14	1.65	13	1.86	13	1.71	14	1.72
Complications of surgical procedures or medical care	14	1.68	14	1.71	14	1.72	15	1.57	15	1.79	14	1.69	16	1.57
Fluid and electrolyte disorders	15	1.67	13	1.93	13	1.73	13	1.70	16	1.56	16	1.50	15	1.60
Rehabilitation care; fitting of prostheses; and adjustment of devices	16	1.64	17	1.46	16	1.60	17	1.49	14	1.81	15	1.69	13	1.74
Complication of device; implant or graft	17	1.46	16	1.60	19	1.40	16	1.51	18	1.34	17	1.47	18	1.42
Acute myocardial infarction	18	1.38	20	1.35	17	1.53	18	1.41	17	1.35	18	1.35	17	1.45
Respiratory failure; insufficiency; arrest (adult)	19	1.34	18	1.45	18	1.42	19	1.41	21	1.21	20	1.30	20	1.36

©2018 Bambhroliya AB et al. *JAMA Network Open*

Description	2010-2015Q3		2010		2011		2012		2013		2014		2015	
	Rank	%	Rank	%	Rank	%	Rank	%	Rank	%	Rank	%	Rank	%
Epilepsy; convulsions	20	1.34	22	1.34	21	1.32	20	1.33	19	1.33	19	1.32	19	1.39
Diabetes mellitus with complications	21	1.23	21	1.35	22	1.18	22	1.20	22	1.15	21	1.29	21	1.20
Hypertension with complications and secondary hypertension	22	1.18	23	1.10	23	1.07	23	1.20	20	1.26	22	1.27	22	1.17
Nonspecific chest pain	23	1.13	19	1.44	20	1.37	21	1.32	23	0.92	24	0.87	24	0.87
Pulmonary heart disease	24	1.00	24	1.06	24	0.98	24	1.03	24	0.92	23	0.95	23	1.01
Coronary atherosclerosis and other heart disease	25	0.87	26	1.02	25	0.96	26	0.99	25	0.82	28	0.71	29	0.66

©2018 Bambhroliya AB et al. *JAMA Network Open*

eTable 5. Top 25 Causes of 30-Day Readmission for Ischemic Stroke by Year

Description	2010-2015Q3		2010		2011		2012		2013		2014		2015	
	Rank	%	Rank	%	Rank	%	Rank	%	Rank	%	Rank	%	Rank	%
Acute cerebrovascular disease	1	20.21	1	18.58	1	19.29	1	19.87	1	20.99	1	20.99	1	21.3
Septicemia (except in labor)	2	8.27	2	6.89	2	7.60	2	7.46	2	8.15	2	9.40	2	9.96
Occlusion or stenosis of pre-cerebral arteries	3	5.13	3	4.83	3	4.87	3	5.07	3	5.51	3	5.46	3	5.05
Acute and unspecified renal failure	4	3.20	7	2.97	5	3.04	6	3.00	4	3.38	4	3.35	4	3.32
Cardiac dysrhythmias	5	2.98	6	3.19	8	2.84	4	3.14	5	3.11	7	2.68	6	2.81
Urinary tract infections	6	2.96	4	3.40	4	3.22	5	3.14	7	2.61	6	2.77	7	2.60
Congestive heart failure; non-hypertensive	7	2.85	8	2.89	6	2.97	8	2.78	6	2.79	5	2.92	5	2.85
Aspiration pneumonitis; food/vomitus	8	2.72	5	3.34	7	2.97	9	2.75	9	2.50	10	2.30	9	2.38
Transient cerebral ischemia	9	2.45	9	2.57	10	2.51	10	2.74	12	2.33	11	2.29	11	2.27
Late effects of cerebrovascular disease	10	2.42	11	2.30	9	2.52	11	2.52	11	2.34	8	2.57	10	2.37
Pneumonia (except that caused by tuberculosis or sexually transmitted disease)	11	2.36	10	2.46	11	2.35	7	2.79	10	2.35	12	2.12	12	2.08
Gastrointestinal hemorrhage	12	2.33	12	2.25	12	2.29	12	2.18	8	2.52	9	2.48	8	2.41
Fluid and electrolyte disorders	13	1.65	13	1.84	13	1.80	13	1.71	16	1.56	15	1.47	15	1.52
Other nervous system disorders	14	1.62	14	1.54	15	1.62	14	1.60	13	1.78	13	1.57	13	1.67
Acute myocardial infarction	15	1.50	18	1.42	14	1.64	16	1.50	17	1.47	16	1.46	16	1.48
Complications of surgical procedures or medical care	16	1.49	15	1.50	16	1.52	17	1.43	15	1.59	14	1.55	17	1.35
Rehabilitation care; fitting of prostheses; and adjustment of devices	17	1.45	21	1.33	19	1.40	20	1.33	14	1.66	17	1.44	14	1.58
Complication of device; implant or graft	18	1.37	17	1.48	20	1.33	15	1.51	19	1.21	18	1.39	19	1.26
Respiratory failure; insufficiency; arrest (adult)	19	1.32	20	1.37	18	1.40	19	1.39	20	1.21	21	1.26	18	1.30

©2018 Bambhroliya AB et al. *JAMA Network Open*

Description	2010-2015Q3		2010		2011		2012		2013		2014		2015	
	Rank	%	Rank	%	Rank	%	Rank	%	Rank	%	Rank	%	Rank	%
Diabetes mellitus with complications	20	1.28	19	1.41	21	1.23	21	1.30	18	1.22	19	1.32	20	1.22
Nonspecific chest pain	21	1.18	16	1.48	17	1.48	18	1.40	23	1.01	23	0.93	23	0.90
Epilepsy; convulsions	22	1.17	22	1.21	22	1.19	22	1.19	22	1.16	20	1.26	21	1.22
Hypertension with complications and secondary hypertension	23	1.10	25	1.04	23	1.08	23	1.11	21	1.17	22	1.20	22	1.08
Coronary atherosclerosis and other heart disease	24	0.94	23	1.09	24	1.07	24	1.09	24	0.93	25	0.79	26	0.71
Syncope	25	0.87	24	1.09	25	0.99	25	1.00	27	0.77	32	0.66	28	0.66

©2018 Bambhroliya AB et al. *JAMA Network Open*

eTable 6. Top 25 Causes of 30-Day Readmission for Intracerebral Hemorrhage by Year

Description	2010-2015Q3		2010		2011		2012		2013		2014		2015	
	Rank	%	Rank	%	Rank	%	Rank	%	Rank	%	Rank	%	Rank	%
Acute cerebrovascular disease	1	17.36	1	16.75	1	17.44	1	16.74	1	16.94	1	18.61	1	17.09
Septicemia (except in labor)	2	11.71	2	10.59	2	9.81	2	11.47	2	12.66	2	13.26	2	13.38
Urinary tract infections	3	3.74	4	3.85	3	4.67	3	3.57	4	3.48	4	3.27	4	3.20
Acute and unspecified renal failure	4	3.25	13	2.06	6	3.43	4	3.18	3	3.53	5	2.94	3	4.16
Aspiration pneumonitis; food/vomitus	5	3.04	3	4.06	5	3.53	7	2.69	5	2.77	7	2.60	6	2.92
Rehabilitation care; fitting of prostheses; and adjustment of devices	6	2.87	6	2.62	8	3.11	9	2.36	6	2.67	3	3.46	5	3.04
Pneumonia (except that caused by tuberculosis or sexually transmitted disease)	7	2.83	5	3.08	4	3.63	6	2.85	11	2.37	10	2.32	9	2.64
Complications of surgical procedures or medical care	8	2.49	10	2.21	7	3.37	13	1.92	10	2.42	8	2.56	7	2.75
Late effects of cerebrovascular disease	9	2.38	11	2.11	10	2.44	5	2.91	9	2.52	9	2.51	11	2.25
Other nervous system disorders	10	2.35	14	2.00	11	2.08	14	1.87	7	2.57	6	2.60	8	2.64
Epilepsy; convulsions	11	2.34	18	1.80	9	2.70	11	2.09	8	2.52	11	2.18	10	2.47
Complication of device; implant or graft	12	1.94	9	2.21	17	1.45	16	1.76	12	2.17	14	1.94	12	2.19
Pulmonary heart disease	13	1.87	12	2.06	12	1.76	10	2.31	16	1.61	15	1.85	18	1.69
Hypertension with complications and secondary hypertension	14	1.86	15	1.95	21	1.09	12	1.92	13	1.97	12	2.13	15	1.85
Fluid and electrolyte disorders	15	1.78	7	2.47	14	1.56	18	1.48	18	1.56	17	1.70	13	2.08
Congestive heart failure; non-hypertensive	16	1.73	19	1.75	19	1.35	8	2.52	15	1.77	21	1.33	20	1.41
Phlebitis; thrombophlebitis and thromboembolism	17	1.73	17	1.85	13	1.76	15	1.87	22	1.16	13	1.99	21	1.29
Cardiac dysrhythmias	18	1.64	8	2.21	15	1.50	17	1.70	17	1.61	20	1.37	17	1.74
Respiratory failure;	19	1.58	16	1.90	18	1.40	20	1.37	20	1.31	18	1.61	16	1.80

©2018 Bambhroliya AB et al. *JAMA Network Open*

Description	2010-2015Q3		2010		2011		2012		2013		2014		2015	
	Rank	%	Rank	%	Rank	%	Rank	%	Rank	%	Rank	%	Rank	%
insufficiency; arrest (adult)														
Secondary malignancies	20	1.54	20	1.18	16	1.45	19	1.43	14	1.82	16	1.70	14	1.91
Gastrointestinal hemorrhage	21	1.31	25	1.03	22	1.09	25	0.88	19	1.51	19	1.61	19	1.57
Residual codes; unclassified	22	0.89	29	0.92	20	1.19	26	0.88	28	0.76	28	0.66	24	0.96
Intracranial injury	23	0.88	31	0.72	27	0.83	21	1.26	27	0.76	23	1.14	22	1.12
Essential hypertension	24	0.86	23	1.08	38	0.57	27	0.82	23	1.11	24	0.95	27	0.79
Diabetes mellitus with complications	25	0.84	22	1.08	29	0.73	30	0.71	34	0.61	22	1.14	29	0.73

©2018 Bambhroliya AB et al. *JAMA Network Open*

eTable 7. Top 25 Causes of 30-Day Readmission for Subarachnoid Hemorrhage by Year

Description	2010-2015Q3		2010		2011		2012		2013		2014		2015	
	Rank	%	Rank	%	Rank	%	Rank	%	Rank	%	Rank	%	Rank	%
Acute cerebrovascular disease	1	16.78	1	16.12	1	17.16	1	17.08	1	15.49	1	18.68	1	15.82
Septicemia (except in labor)	2	7.69	4	4.03	3	5.77	2	8.78	2	7.82	2	8.43	2	11.05
Other hereditary and degenerative nervous system conditions	3	5.79	3	4.19	2	6.55	3	5.80	3	6.64	3	6.88	4	3.91
Complications of surgical procedures or medical care	4	4.59	2	5.58	4	3.74	4	5.33	4	4.42	4	3.65	3	4.76
Headache; including migraine	5	3.18	5	3.57	9	2.96	6	3.29	7	2.95	6	3.09	6	3.40
Other and ill-defined cerebrovascular disease	6	3.15	6	3.57	7	3.12	8	2.66	11	2.65	5	3.51	7	2.89
Rehabilitation care; fitting of prostheses; and adjustment of devices	7	2.79	15	2.02	8	3.12	9	2.66	5	3.54	8	2.67	8	2.72
Urinary tract infections	8	2.69	11	2.64	6	3.28	5	3.61	6	3.10	12	2.11	15	1.70
Other nervous system disorders	9	2.56	10	2.79	14	2.18	13	2.35	8	2.80	7	2.67	13	2.04
Complication of device; implant or graft	10	2.56	12	2.48	5	3.59	12	2.51	10	2.80	14	2.11	12	2.21
Epilepsy; convulsions	11	2.49	9	2.79	15	2.03	7	2.66	12	2.36	18	1.54	5	3.57
Transient cerebral ischemia	12	2.49	8	2.95	10	2.81	10	2.51	14	2.06	10	2.25	9	2.38
Pneumonia (except that caused by tuberculosis or sexually transmitted disease)	13	2.05	7	3.10	12	2.65	11	2.51	20	1.47	16	1.69	23	1.19
Cardiac and circulatory congenital anomalies	14	2.02	16	1.86	13	2.50	16	1.88	17	1.92	9	2.53	20	1.36
Pulmonary heart disease	15	1.79	13	2.17	16	1.72	20	1.25	9	2.80	19	1.54	14	1.70
Late effects of cerebrovascular disease	16	1.79	14	2.02	17	1.56	24	0.94	16	1.92	15	1.97	10	2.38
Phlebitis; thrombophlebitis and thromboembolism	17	1.79	21	1.40	11	2.81	17	1.72	15	2.06	20	1.40	19	1.36
Fluid and electrolyte disorders	18	1.49	19	1.40	30	0.78	14	2.04	19	1.47	17	1.54	16	1.53
Acute and unspecified renal failure	19	1.46	23	1.40	27	1.09	25	0.94	18	1.77	11	2.25	17	1.53

©2018 Bambhroliya AB et al. *JAMA Network Open*

Description	2010-2015Q3		2010		2011		2012		2013		2014		2015	
	**Rank**	**%**	**Rank**	**%**	**Rank**	**%**	**Rank**	**%**	**Rank**	**%**	**Rank**	**%**	**Rank**	**%**
Intracranial injury	20	1.43	26	1.24	21	1.25	43	0.47	13	2.21	13	2.11	25	1.02
Respiratory failure; insufficiency; arrest (adult)	21	1.15	29	0.93	26	1.09	19	1.41	26	0.88	39	0.56	11	2.21
Aspiration pneumonitis; food/vomitus	22	1.08	22	1.40	25	1.09	15	1.88	25	1.03	28	0.84	33	0.68
Congestive heart failure; non-hypertensive	23	1.05	24	1.24	18	1.40	27	0.78	21	1.18	27	0.84	31	0.68
Hypertension with complications and secondary hypertension	24	1.02	20	1.40	47	0.31	22	1.10	24	1.03	22	1.12	18	1.36
Meningitis (except that caused by tuberculosis or sexually transmitted disease)	25	1.00	17	1.71	23	1.09	21	1.10	29	0.74	25	0.84	41	0.51

©2018 Bambhroliya AB et al. *JAMA Network Open*

eTable 8. Comparison of Length of Stay, In-hospital Mortality, and Cost of Care for Ischemic and Hemorrhagic Stroke by Year

	**Acute Ischemic Stroke**		**Intracerebral Hemorrhage**		**Sub Arachnoid Hemorrhage**	
	**Index Stay**	**30-Day Readmission**	**Index Stay**	**30-Day Readmission**	**Index Stay**	**30-Day Readmission**
LOS (mean, SE)	4.92 (0.02)	6.50 (0.03)	7.64 (0.05)	7.82 (0.11)	11.64 (0.10)	7.50 (0.19)
Mortality (%, 95% CI)	5.13 (5.05 5.22)	6.54 (6.36-6.73)	28.68 (28.30-29.07)	7.65 (7.09-8.25)	21.95 (21.35-22.56)	4.64 (3.92-5.48)
Cost / stay (mean, $2014)	10881	12303	21154	14377	51032	17340
Cost / day (mean, $2014)	2696	2392	2422	2191	3978	2990
**Cost / stay by year (mean, $2014)**						
2010	9355	10585	17806	12665	42262	14590
2011	10327	11957	20341	12766	48022	15818
2012	10909	12222	21753	13822	51598	18669
2013	11536	13272	22059	15568	55329	17390
2014	11950	13387	23371	16881	56286	20020
**Cost / day by year (mean, $2014)**						
2010	2198	2054	2037	1871	3351	2431
2011	2434	2225	2239	2027	3686	2736
2012	2694	2408	2461	2253	3910	2968
2013	2951	2602	2594	2334	4296	3241
2014	3089	2652	2717	2458	4501	3516

Cost were inflation adjusted using Chained Consumer Price Index (CPI) for All Urban Consumers – Medical Care Services from the U.S. Bureau of Labor Statistics (https://www.bls.gov/cpi/data.htm) and reported as 2014 average dollar. Mean length of stay (LOS) and mortality rate were calculated using survey methods. All discharge types were included in calculating Index stay numbers for LOS and mortality rate.LOS, length of stay; SE, standard error; CI, confidence interval.

©2018 Bambhroliya AB et al. *JAMA Network Open*

eFigure. Probability and 95% CI (Y-Axis) for 30-Day Stroke Related Readmissions for Hospitals With Varying Stroke Discharge Volume (X-Axis) Based on Hospitals' Teaching Status

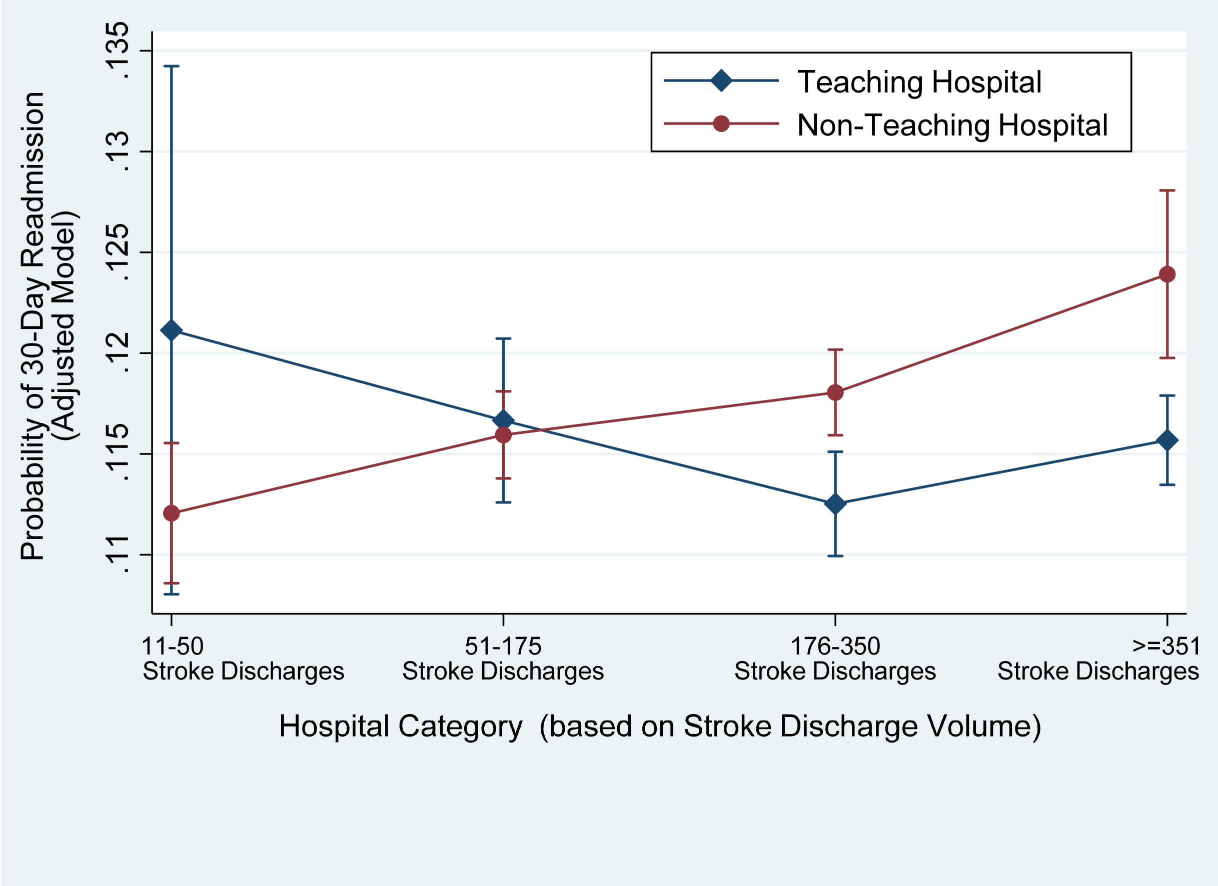

©2018 Bambhroliya AB et al. *JAMA Network Open*

eReferences

1. Centers for Disease Control and Prevention. International Classification of Diseases, Ninth Revision, Clinical Modification (ICD-9-CM). http://www.cdc.gov/nchs/icd/icd9cm.htm. Accessed December 1, 2017.
2. Tirschwell DL, Longstreth WT, Jr. Validating administrative data in stroke research. *Stroke.* 2002;33(10):2465-2470.
3. Roumie CL, Mitchel E, Gideon PS, Varas-Lorenzo C, Castellsague J, Griffin MR. Validation of ICD-9 codes with a high positive predictive value for incident strokes resulting in hospitalization using Medicaid health data. *Pharmacoepidemiol Drug Saf.* 2008;17(1):20-26.
4. Kokotailo RA, Hill MD. Coding of stroke and stroke risk factors using international classification of diseases, revisions 9 and 10. *Stroke.* 2005;36(8):1776-1781.
5. Thigpen JL, Dillon C, Forster KB, et al. Validity of international classification of disease codes to identify ischemic stroke and intracranial hemorrhage among individuals with associated diagnosis of atrial fibrillation. *Circ Cardiovasc Qual Outcomes.* 2015;8(1):8-14.
6. Cunningham A, Stein CM, Chung CP, Daugherty JR, Smalley WE, Ray WA. An automated database case definition for serious bleeding related to oral anticoagulant use. *Pharmacoepidemiol Drug Saf.* 2011;20(6):560-566.
7. Center for Medicare & Medicaid Services. 2014 measure information about the 30-day all-cause hospital readmission measure, calculated for the value-based payment modifier program. https://www.cms.gov/Medicare/Medicare-Fee-for-Service-Payment/PhysicianFeedbackProgram/Downloads/2014-ACR-MIF.pdf. Last Updated December 2016. Published April 2015. Accessed April 26, 2018
8. Prevention Quality Indicators Overview. Agency for Healthcare Research and Quality. http://www.qualityindicators.ahrq.gov/Modules/pqi_resources.aspx. Accessed December 1, 2017.
9. Qureshi AI, Harris-Lane P, Siddiqi F, Kirmani JF. International classification of diseases and current procedural terminology codes underestimated thrombolytic use for ischemic stroke. *J Clin Epidemiol.* 2006;59(8):856-858.
10. Moradiya Y, Crystal H, Valsamis H, Levine SR. Thrombolytic utilization for ischemic stroke in US hospitals with neurology residency program. *Neurology.* 2013;81(23):1986-1995.
11. Dasenbrock HH, Cote DJ, Pompeu Y, Vasudeva VS, Smith TR, Gormley WB. Validation of an International Classification of Disease, Ninth Revision coding algorithm to identify decompressive craniectomy for stroke. *BMC Neurol.* 2017;17(1):121.
12. Quan H, Parsons GA, Ghali WA. Validity of procedure codes in International Classification of Diseases, 9th revision, clinical modification administrative data. *Med Care.* 2004;42(8):801-809.
13. Chio A, Ciccone G, Calvo A, et al. Validity of hospital morbidity records for amyotrophic lateral sclerosis. A population-based study. *J Clin Epidemiol.* 2002;55(7):723-727.
14. RAND Corporation. Evaluations of severity-adjusted DRG systems: report WR-434-CMS. www.rand.org/pubs/working_papers/WR434/. Published July 29, 2007. Accessed April 26, 2018.
15. Moradiya Y, Murthy SB, Newman-Toker DE, Hanley DF, Ziai WC. Intraventricular thrombolysis in intracerebral hemorrhage requiring ventriculostomy: a decade-long real-world experience. *Stroke.* 2014;45(9):2629-2635.
